# TM7SF2 as a Potential Biomarker in Colorectal Cancer: Implications for Metastasis

**DOI:** 10.3390/curroncol32020114

**Published:** 2025-02-17

**Authors:** Inpyo Hong, Sooyoun Kim, Minho Lee, Seoin Han, Hak Chun Kim, Chong Woo Chu, Seong Geun Kim, Min Kyung Kim, Chang Jin Kim, Dong Hyun Kang, Tae Sung Ahn, Moo Jun Baek, Mudasir Hussain, Hyog Young Kwon, Dongjun Jeong

**Affiliations:** 1 Department of Pathology, College of Medicine, Soonchunhyang University, Dongnam-gu, Cheonan 31151, Republic of Korea; hip0725@sch.ac.kr (I.H.); sooy.kim@sch.ac.kr (S.K.); leemho00@sch.ac.kr (M.L.); gkstjdls00@sch.ac.kr (S.H.); 2 Soonchunhyang Medical Science Research Institute, College of Medicine, Soonchunhyang University, Dongnam-gu, Cheonan 31151, Republic of Korea; khc5467@sch.ac.kr; 3 Changwon Hanmaeum Hospital, Uichang-gu, Changwon-si 51139, Republic of Korea; liversurgeon@hanmail.net (C.W.C.); sgkimpatheny@gmail.com (S.G.K.); minkyungk76@naver.com (M.K.K.); mountain48@hanmail.net (C.J.K.); 4 Department of Surgery, College of Medicine, Soonchunhyang University, Dongnam-gu, Cheonan 31151, Republic of Korea; c100048@schmc.ac.kr (D.H.K.); eyetoeye@schmc.ac.kr (T.S.A.); ssurge@sch.ac.kr (M.J.B.); 5 Department of Integrated Biomedical Science, Soonchunhyang University, Cheonan-si 31151, Republic of Korea; khaylihussain@gmail.com (M.H.); hykwon@sch.ac.kr (H.Y.K.)

**Keywords:** TM7SF2, colorectal cancer, prognostic biomarker, therapeutic target, biomarker

## Abstract

Colorectal cancer (CRC) is a commonly fatal cancer and ranks as the fourth most prevalent in men and third in women worldwide. While early-stage survival rates are high, they significantly decrease with recurrence and metastasis. Thus, the early detection and treatment of metastasis-related factors can significantly improve survival rates. In this study, the transmembrane 7 superfamily member 2 (TM7SF2) gene was validated as a biomarker for predicting metastasis in CRC. Immunohistochemical staining was performed on 236 CRC tissues, and the clinicopathological factors of patients with CRC were analyzed. This evaluation revealed that TM7SF2 expression is associated with the clinical stage. Kaplan–Meier analysis confirmed the relationship between the survival rate of CRC patients and TM7SF2 expression, showing a decrease in survival rate with TM7SF2 overexpression (log-rank, *p* < 0.001). TM7SF2 expression was also confirmed in two pairs of primary and metastatic cell lines (SW480 and SW620). TM7SF2 knockdown was executed using siRNAs in SW480 and SW620 cells, which exhibit high expression levels. The knockdown was verified using RT-PCR and immunoblotting. Functional studies investigated the effects of TM7SF2 on cell proliferation, migration, invasion, and colony formation, revealing that all these functions were suppressed in the CRC cell lines following TM7SF2 knockdown. Therefore, TM7SF2 shows promise as a biomarker for the prevention of colorectal cancer.

## 1. Introduction

The large intestine, a key digestive organ, comprises the colon, rectum, and anal canal. Colorectal cancer (CRC) is a malignancy that develops in the epithelium of the colon and rectum. The occurrence of CRC is based on location: 50% in the rectum, 49% in the colon, and 1% in both sites. The cancers predominantly occur in the sigmoid colon (54%), ascending colon (22%), transverse colon (8%), descending colon (7%), cecum (7%), and at crossing sites (2%) [1]. According to a report by the American Cancer Society (ACS) [2], CRC has the third-highest incidence and the second-highest mortality rate among cancers globally. Statistics Korea and the National Cancer Information Center report that, as of 2022, the mortality rate due to cancer in the Republic of Korea is 22.8% of the total death rate, with CRC being the third most common cancer, having an incidence rate of 11.2% [3,4]. Moreover, the survival or mortality of CRC patients is closely linked to the presence or absence of distant metastases. As reported by the National Cancer Information Center of the Republic of Korea, the 5-year survival rate for patients without metastasis is 94%. However, this rate drops to 82.5% in cases of regional metastasis and further decreases to 20% in cases of distant metastasis [4]. Consequently, there is a critical need for prognostic biomarkers to improve survival rates in patients with CRC.

Transmembrane 7 superfamily member 2 (TM7SF2), also known as DHCR14A or ANG1, is a gene located in chromosome 11q13.1 that encodes Δ14-sterol reductase, an enzyme involved in cholesterol biosynthesis [5]. The TM7SF2 gene is expressed in normal tissues of the pancreas, brain, heart, lung, kidney, ovary, testis, prostate, and skeletal muscle but not in the colon, small intestine, spleen, placenta, thymus, or leukocytes [6,7]. Intriguingly, an investigation using the GEPIA database (http://gepia2.cancer-pku.cn/ (accessed on 18 November 2023)) revealed that TM7SF2 expression is upregulated in various cancer tissues compared to normal tissues. Additionally, TM7SF2 has been implicated in a range of human diseases, such as Greenberg’s dysplasia, CK syndrome, skin papilloma, and cervical cancer, highlighting its importance in physiological regulation and pathological diagnosis [8,9,10,11,12,13].

According to other researchers, gene expression profiling has revealed variations in TM7SF2 expression between nonaggressive and aggressive follicular carcinomas. This finding suggests that TM7SF2 could serve as a valuable prognostic biomarker for distinguishing these types of cancer [14]. Studies involving TM7SF2 knockout mice have shown that these mice experience a delay in cell cycle progression at the G1/S phase during liver regeneration, which results in slower cell division rates [15]. Furthermore, these mice exhibit a significant increase in the expression of the tumor suppressor p53 following partial hepatectomy [15].

In the context of cervical cancer, recent findings indicate a link between the activation of the TM7SF2/C-Raf/MEK/ERK pathway and an increase in cell proliferation and migration [16]. This is particularly noteworthy as the dysregulation of the extracellular signal-regulated kinase (ERK) and mitogen-activated protein kinase (MAPK) pathways is a hallmark of various human cancers, influencing cell proliferation, migration, and angiogenesis through the activation of the ERK1/2 pathway via phosphorylation [17,18].

The Raf kinases (Raf), comprising A-Raf, B-Raf, and C-Raf subtypes, each with a Ras-binding domain at their *N*-terminal region, play a crucial role in this process [19,20]. Raf, a member of the protein serine/threonine kinase family, is responsible for catalyzing the phosphorylation and subsequent activation of MEK1/2, which further activates ERK [21]. Despite these insights, the specific role of TM7SF2 in colorectal cancer remains an area for further exploration and research.

This study investigates the correlation between TM7SF2 expression and the prognosis of colorectal cancer patients. Furthermore, we demonstrate that the downregulation of TM7SF2 in colorectal cancer cell lines inhibits key cancer cell functions, highlighting its potential role as a therapeutic target.

## 2. Materials and Methods

### 2.1. Tissue Samples

A tissue microarray (TMA), including 236 cases of colorectal cancer (CRC), 88 cases of metastasis, and nine cases of adjacent normal colorectal tissues, was purchased from SuperBioChips, TissueARRAY (Seoul, Republic of Korea, https://www.tissue-array.com/), to evaluate the expression of TM7SF2 in colorectal cancer tissues. The patient information is presented in Table 1.

### 2.2. Immunohistochemistry Staining

The TMA slide was deparaffinized at 60 °C for 1 h, immersed in xylene 3 times, and then hydrated in 100% ethanol to 70% ethanol for 3 min each. Antigen retrieval was performed in 10 mM Tris and 1 mM EDTA solution (pH 9.0) (Invitrogen, Waltham, MA, USA, #00-4956-58) by heating for 10 min using a microwave. The slide was incubated overnight at 4 °C with antihuman TM7SF2 polyclonal rabbit antibody (Proteintech, IL, USA, 1:100) diluted in 1% BSA solution. Next, the slide was washed three times with PBST, and antirabbit HRP-conjugated secondary antibody (Thermo Fisher Scientific, Waltham, MA, USA, 1:100) was added for 4 h at 23 °C. The slides were then stained with diaminobenzidine (DAB, Vector, CA, USA) as a brown chromogen, and the nuclei were counterstained with hematoxylin. Each core on the slide was scored by multiplying the percentage and intensity by grades. The evaluation of percentages is as follows: 0 points (0–10%), 1 point (10–30%), 2 points (30–50%), and 4 points (70–100%). The intensity was classified as follows: 0 (negative), 1 (weak), 2 (moderate), and 3 (strong). In the final multiplication score, a value < 4 was classified as low expression, and a value ≥ 4 was classified as high expression.

### 2.3. Cell Culture

Human CRC cell lines (SW480 and SW620) were purchased from the Korea Cell Line Bank (KCLB, Seoul, Republic of Korea). Each cell lines were cultured in RPMI1640 (Hyclone, Logan, UT, USA) containing 10% Fetal bovine serum (FBS, Thermo Fisher Scientific, MA, USA), 100 U/mL penicillin, and 100 ug/mL streptomycin (P/S, Hyclone, UT, USA) at 37 °C in an environment at 5% CO_2_. Cells were maintained in culture dishes and passaged every 3 days with 0.25% trypsin (Hyclone, UT, USA).

### 2.4. Gene Knockdown by Small Interfering RNA (siRNA)

TM7SF2 knockdown was achieved in colorectal cancer cell lines SW480 and SW620 using siRNA transfection. All siRNAs targeting human TM7SF2 were pooled before use. The siRNAs used were as follows: TM7SF2-siRNA 1, TM7SF2-siRNA 2, and TM7SF2-siRNA 3, provided by Bioneer, Daejeon, Republic of Korea. The sequences for these siRNAs are TM7SF2-siRNA 1 Forward GUG UUU CCU UGA CUG ACU A, TM7SF2-siRNA 1 Reverse UAG UCA GUC AAG GAA ACA C, TM7SF2-siRNA 2 Forward GAC AGU GUU UCC UUG ACU G, TM7SF2-siRNA 2 Reverse CAG UCA AGG AAA CAC UGU C, TM7SF2-siRNA 3 Forward CGU AUC UGU UUC UUC GAC U, TM7SF2-siRNA 3 Reverse AGU CGA AGA AAC AGA UAC G. These siRNAs were pooled and diluted to a final concentration of 100 nM in 150 µL of Opti-MEM (Thermo Fisher, Waltham, MA, USA). Concurrently, 9 µL of Lipofectamine RNAiMAX (Thermo Fisher, MA, USA) was also diluted in 150 µL of Opti-MEM. The diluted siRNAs and Lipofectamine RNAiMAX were mixed to form an siRNA-lipid complex and incubated for 5 min at room temperature. The colorectal cancer cell lines were seeded at 2.5 × 10^5^ cells/well in 6-well plates and grown to 30–70% confluence in RPMI1640 medium supplemented with 10% FBS and without P/S. Before transfection, the culture medium was replaced with a serum-free medium. The prepared siRNA-lipid complex was then added to each well. The plates were gently shaken and incubated at 37 °C in a 5% CO_2_ atmosphere. This methodology was applied to both the TM7SF2 knockdown group and the negative control group, which was treated with Accutarget™ Negative Control siRNA (Bioneer, Daejeon, Republic of Korea).

### 2.5. RNA Extraction and Real-Time Polymerase Chain Reaction

Two groups of cell lines (negative control and TM7SF2 knockdown) were washed with PBS, and total RNA was extracted using 1 mL of RiboEx solution (Geneall, Seoul, Republic of Korea) and a Hybrid-RTM Kit (Geneall, Seoul, Republic of Korea). The extracted total RNA was measured using a Microvolume Spectrophotometer (Pulton, USA) and quantified at 500 ng. cDNA was synthesized using the ReverTra Ace qPCR RT kit (TOYOBO, Osaka, Japan). To confirm whether TM7SF2 was suppressed in the cell lines, real-time RT-PCR was performed using the SYBR Green Real-time PCR Master Mix (TOYOBO, Osaka, Japan). Primer sequences are TM7SF2 forward 5′-GGTCAATGGCTTCCAGTTGCT-3′ reverse 5′-AACGCCAGCATGAAGCCAAACC-3′, and GAPDH forward 5′-GTCTCCTCTGACTTCAACAGCG-3′ reverse 5′-ACCACCCTGTTGCTGTAGCCAA-3′. The PCR parameters were as follows: after pre-denaturation (95 °C, 10 min), 30 cycles of denaturation (95 °C, 30 s), annealing (57 °C, 15 s), and extension (72 °C, 15 s). The expression of each mRNA was standardized to that of glyceraldehyde-3-phosphate dehydrogenase (GAPDH).

### 2.6. Western Blot

Two groups of cell lines (negative control and TM7SF2 knockdown) were each lysed in RIPA Lysis and Extraction Buffer (Thermo Fisher Scientific, MA, USA), and then centrifuged at 12,000× *g* for 20 min at 4 °C. Protein samples were aligned on a stacking gel at 70 V for 20 min and electrophoresed on a separating gel at 100 V for 100 min. Protein was transferred to a 0.2 um polyvinylidene difluoride membrane (PVDF, Merck Millipore, Burlington, MA, USA). The membrane was blocked with 5% BSA solution for 1 h and incubated with antihuman TM7SF2 polyclonal rabbit antibody (Proteintech, Rosemont, IL, USA, 1:1000) overnight at 4 °C. The next day, the membrane was then washed with TBST and incubated with antirabbit HRP-conjugated secondary antibody (Thermo Fisher Scientific, MA, USA, 1:100) for 2 h at 23 °C. After the reaction, proteins were visualized using PierceTM ECL Western Blotting Substrate (Thermo Fisher Scientific, MA, USA) and a ChemiDoc XRP + system (Bio-Rad, Hercules, CA, USA). The relative expression of each protein was normalized to ß-actin using Image J (https://imagej.net/ij/).

### 2.7. Cell Proliferation Assay (WST-1)

Cell proliferation was measured in two groups of cells (negative control and TM7SF2 knockdown) cell lines. Each cell was suspended in RPMI1640 with 10% FBS and 1% P/S and seeded in 96-well plates at a concentration of 1 × 10^4^ cells/well in 100 μL. Then, 10 μL of Cyto X (LPS solution, Daejeon, Republic of Korea) was applied to each well at 24, 48, and 72 h and incubated for 1 h at 37 °C, 5% CO_2_. The absorbance of the samples was measured at 450 nm using a MultiskanTM GO microplate spectrophotometer (Thermo Fisher Scientific, MA, USA).

### 2.8. Migration and Invasion Assay

A tissue microarray (TMA), including 236 cases of colorectal cancer (CRC), 10 cases of metastasis, and nine cases of adjacent normal colorectal tissues, was purchased from SuperBioChips, TissueARRAY (Republic of Korea, https://www.tissue-array.com/), to evaluate the expression of TM7SF2 in colorectal cancer tissues. The patient information is presented in Table 1.

### 2.9. Colony Forming Assay

Agarose 1% was dissolved in PBS using a microwave, and then 1% agarose and RPMI1640 with 20% FBS was adjusted to a temperature of 40 °C in a water bath. Equal volumes of 1% agarose and medium were mixed to obtain a final 0.5% base agarose. Base agarose 0.5% was plated 1.5 mL/well in a 6-well plate. The 0.7% top agar was dissolved in the same way as the base agar and adjusted to a temperature of 40 °C. Also, warmed RPMI1640 with 20% FBS. Then, 0.7% agar was mixed with the medium to make 0.35% agar, and then 10,000 cells/well were added to the 1.5 mL agar mixture. Each cell-mixed agar mixture was then added to the base agar and incubated at 37 °C, 5% CO_2_. After incubation for three weeks, cells were stained with 0.05% crystal violet. The colonies were counted under a microscope.

### 2.10. Statistical Analysis

All data are presented as the average of triplicate measurements. Statistical analysis was performed using SPSS 23.0, and data were presented as mean ± SDM (standard deviation mean). The relationship between various clinicopathological factors and TM7SF2 expression was assessed using the chi-square test. The 5-year survival rate of patients with CRC according to TM7SF2 expression was analyzed using the Kaplan–Meier analysis, and the log-rank test was used to assess statistical significance. Univariate and multivariate Cox regression analyses were used to estimate the risk ratio between clinicopathological factors and genes. All statistical significance was estimated at *p* < 0.05, and the Cox regression risk was assessed with a 95% confidence interval (CI).

## 3. Results

### 3.1. Relationship of TM7SF2 Expression with the Progression of Colorectal Cancer

**Figure 1:**
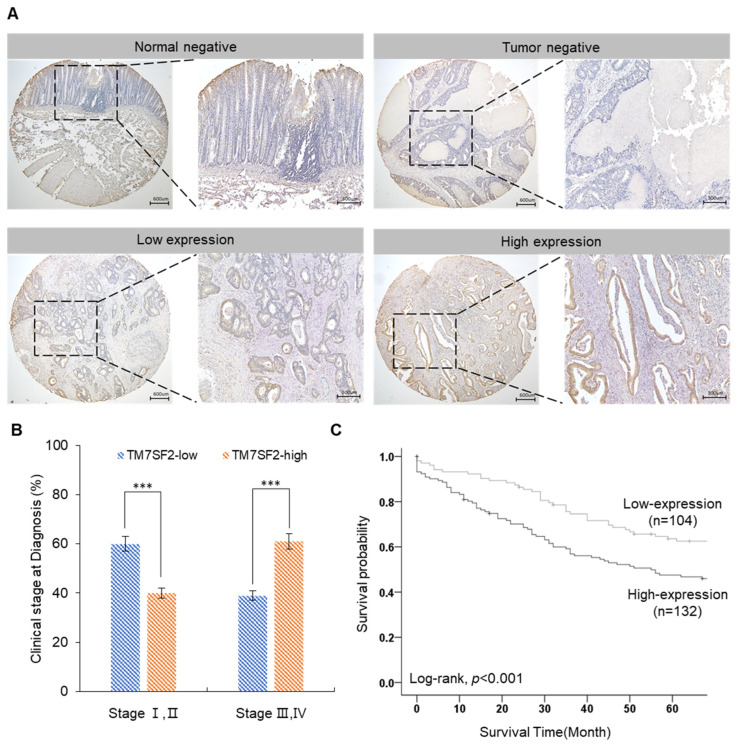
TM7SF2 overexpression and its association with poor prognosis in CRC patients. (**A**) Immunohistochemistry assays demonstrate higher TM7SF2 expression levels in tumor tissues compared to normal (adjacent tumor) tissues. (**B**) A correlation between TM7SF2 overexpression and higher clinical stages in CRC patients (n = 234, *** *p* < 0.001). (**C**) Association of TM7SF2 expression with the overall survival of CRC patients, as illustrated by the Kaplan–Meier curve.

The expression of TM7SF2 was confirmed through immunohistochemistry staining and data analysis. Tissue microarray (TMA) slides, comprising 236 tissues, were stained with a TM7SF2 antibody. It was observed that TM7SF2 was not expressed in normal colon tissues. However, in colorectal cancer tissues, TM7SF2 exhibited strong expression in the plasma membrane and cytoplasm (Figure 1A). Furthermore, in the more advanced stages of the disease (stages III and IV), a higher frequency of TM7SF2 expression was noted compared to the early stages (stages I and II). Specifically, in the lower stages, 60% showed low expression and 40% high expression, while in the higher stages, 38.6% demonstrated low expression and 61.4% high expression (Figure 1B). The association between TM7SF2 expression and 5-year survival rates was analyzed using Kaplan–Meier survival curves. The analysis revealed that approximately 14% of patients with low TM7SF2 expression survived, in contrast to only 4.2% survival in patients with high TM7SF2 expression. These results indicate that colorectal cancer patients with high TM7SF2 expression have a poorer prognosis compared to those with low expression of the protein (log-rank, *p* < 0.001, Figure 1C).

### 3.2. Expression of TM7SF2 Was Associated with Clinicopathological Factors in Colorectal Cancer

High expression of TM7SF2 was detected in 132 out of 236 samples, accounting for 55.9% of the cases. The relationship between TM7SF2 expression and various factors, including age, gender, tumor size (pT stage), lymph node involvement (pN stage), metastasis, and clinical stage, was statistically analyzed (Table 2). These findings suggest that TM7SF2 expression contributes to the metastasis of colorectal cancer (CRC) and is correlated with the clinical stage of the disease. In the early clinical stages (I and II), there was low TM7SF2 expression, and the post clinical stage (III and IV) had high expression of TM7SF2. Furthermore, independent prognostic factors for the 236 colorectal cancer samples were identified using Cox regression analysis. The univariate analysis indicated that the pT stage (*p* = 0.019), pN stage (*p* < 0.001), metastasis (*p* = 0.004), and clinical stage (*p* < 0.001) were significantly associated with outcomes (Table 3). Additionally, multivariate analysis revealed that the clinical stage (*p* < 0.001) was a significant predictor of prognosis (Table 4).

### 3.3. Downregulation of TM7SF2 mRNA and Protein Expression in CRC Cells Using siRNA

**Figure 2:**
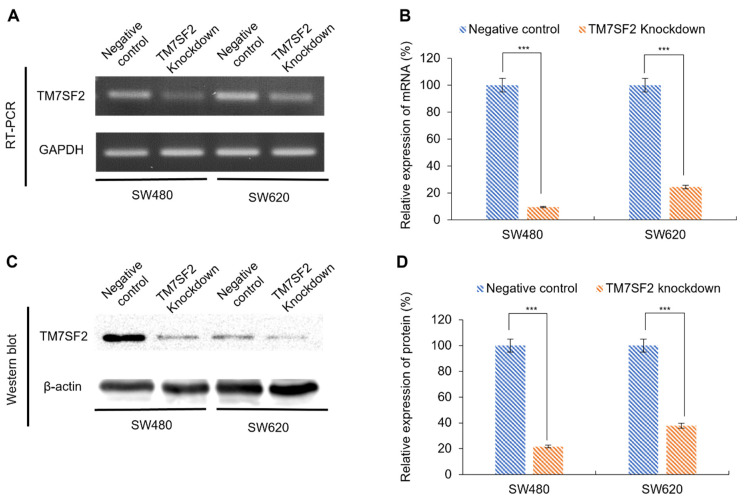
Confirmation of TM7SF2 expression after siRNA transfection through RT-PCR and Western blot. (**A**) mRNA expression levels of TM7SF2 in CRC cells (SW480 and SW620) post-TM7SF2 knockdown, as confirmed by RT-PCR. (**B**) Comparison of TM7SF2 expression in the knockdown group relative to the negative control, normalized using GAPDH. (**C**) Protein expression levels of TM7SF2 in CRC cells (SW480 and SW620) after siRNA transfection, evaluated by Western blot. (**D**) Comparison of TM7SF2 expression in the knockdown group to the negative control, quantified using β-actin (*** *p* < 0.001). The uncropped blots are shown in the Supplemental Materials.

To investigate the impact of reduced TM7SF2 expression on the progression and metastasis of cancer cells, siRNAs were employed to downregulate TM7SF2 in SW480 and SW620 cell lines. TM7SF2 expression was effectively suppressed using siRNA, and the subsequent decrease in mRNA and protein levels was verified through RT-PCR and Western blot analyses. In the TM7SF2 knockdown group, mRNA expression levels were reduced by 90.3% in SW480 cells and 75.5% in SW620 cells, compared to the negative control (Figure 2A,B). Similarly, protein expression levels in the TM7SF2 knockdown group decreased by 78.4% in SW480 cells and 62.2% in SW620 cells relative to the negative control group (Figure 2C,D). Both the negative control and TM7SF2 knockdown groups were subsequently utilized for functional studies.

### 3.4. The Effect of TM7SF2 Downregulation on the Proliferation of CRC Cells

**Figure 3:**
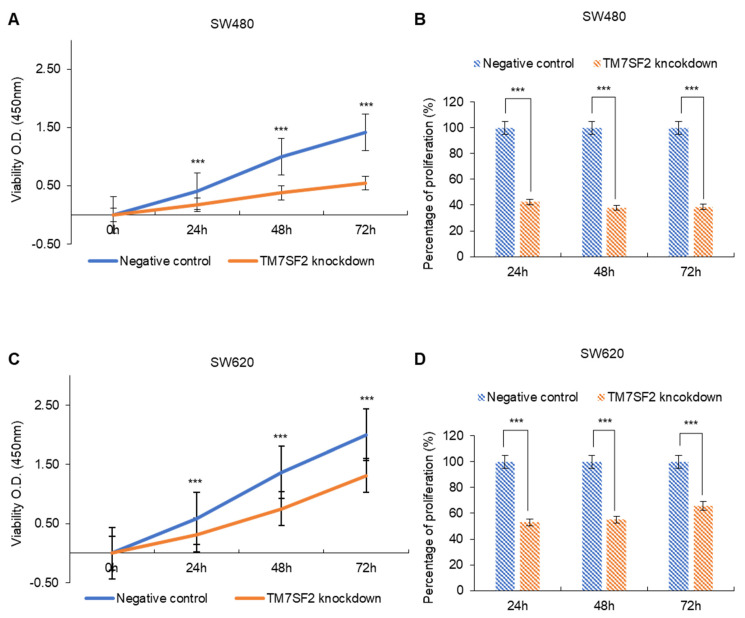
TM7SF2 knockdown reduces the proliferation of CRC cells, as evidenced by the WST-1 assay. (**A**) Measurement of absorbance at 450 nm over time in SW480 cells. (**B**) Comparison of the proliferation rate in the TM7SF2 knockdown group to the SW480 negative control. (**C**) Measurement of absorbance in SW620 cells following WST-1 treatment. (**D**) Comparison of the proliferation rate in the TM7SF2 knockdown group to the SW620 negative control (*** *p* < 0.001).

A WST-1 assay was conducted to examine the impact of TM7SF2 downregulation on the proliferation of colorectal cancer cells. The proliferation rate in the TM7SF2 knockdown group was lower than in the negative control group. In SW480 cells, the proliferation rate of the TM7SF2 knockdown group was reduced to 57.5 ± 1.09% at 24 h, 62 ± 3.2% at 48 h, and 61.4 ± 1.84% at 72 h (Figure 3A,B). In SW620 cells, the proliferation rate was reduced to 47 ± 2.3% at 24 h, 45 ± 4.13% at 48 h, and 34.3 ± 3.41% at 72 h in the TM7SF2 knockdown group (Figure 3C,D). These results demonstrate that the downregulation of TM7SF2 expression inhibits the proliferation of colorectal cancer cells.

### 3.5. The Effect of TM7SF2 Downregulation on the Migration and Invasion of CRC Cells

**Figure 4:**
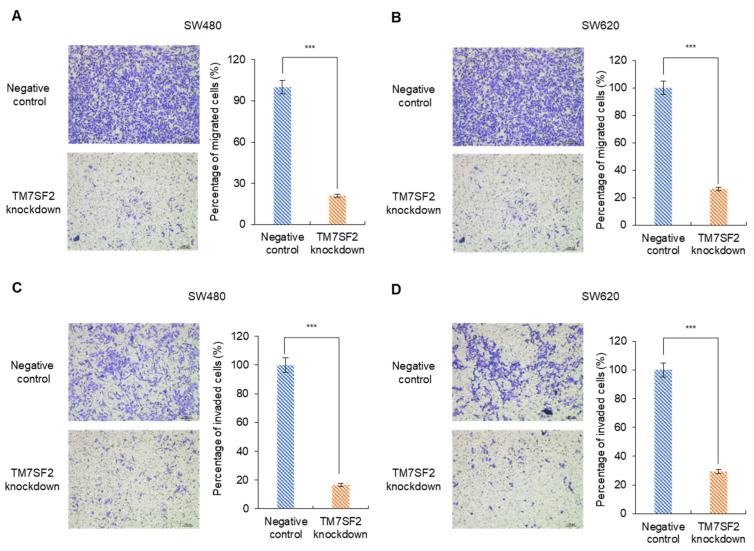
The Transwell analysis demonstrates reduced migration and invasion of CRC cells following TM7SF2 downregulation. (**A**,**B**) Migration of CRC cells to the bottom chamber was inhibited in the TM7SF2 knockdown group compared to the negative controls; (**A**) SW480 and (**B**) SW620. (**C**,**D**) Invasion into the bottom chamber was similarly inhibited in the TM7SF2 knockdown group over the negative controls; (**C**) SW480 and (**D**) SW620. (*** *p* < 0.001, scale bar: 100 μm at 100× magnification).

The migration and invasion abilities of colorectal cancer cells were assessed by comparing the TM7SF2 knockdown group with the negative control group. In the migration assay, the SW480 negative control group exhibited an average cell count of 505, whereas the TM7SF2 knockdown group showed a reduced count of 103, indicating a decrease of 79.6 ± 6.9% (Figure 4A). In the case of SW620, the average cell count in the negative control group was 821, which decreased to 206 in the TM7SF2 knockdown group, representing a reduction of 74.8 ± 8.5% (Figure 4B). Regarding invasion, the average cell count in the SW480 negative control group was 400, compared to 67 in the TM7SF2 knockdown group, a decrease of 83.3 ± 5.3% (Figure 4C). In SW620, the negative control group had an average of 434 cells, while the TM7SF2 knockdown group had 128, showing a decrease of 70.6 ± 13.9% (Figure 4D). These results confirm that TM7SF2 plays a significant role in the migration and invasion of colorectal cancer cells.

### 3.6. Downregulation of TM7SF2 Reduced the Colony-Forming Ability of Colorectal Cancer Cells

**Figure 5:**
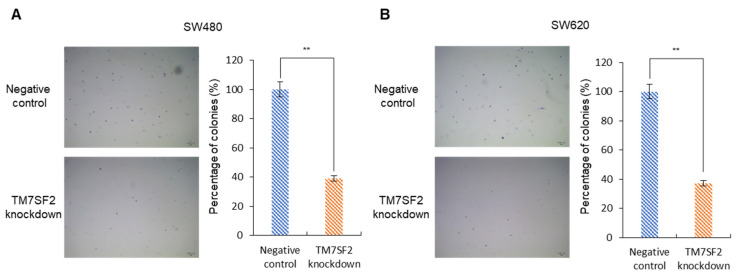
TM7SF2 downregulation decreases colony-formation in CRC cells as demonstrated by the soft agar colony forming assay. (**A**,**B**) Colony formation was inhibited in CRC cells with TM7SF2 knockdown compared to the negative control; (**A**) SW480 and (**B**) SW620. (** *p* < 0.01, Scale bar: 100 μm (40× magnification)).

A soft agar colony-forming assay was conducted to assess the impact of TM7SF2 downregulation on cancer cell colony formation. In this assay, the SW480 negative control group exhibited an average colony count of 52, whereas the TM7SF2 knockdown group had an average of 13, marking a decrease of 60.9 ± 9.3% (*p* < 0.01, Figure 5A). In the case of SW620 cells, the average colony count in the negative control group was 50, compared to 19 in the TM7SF2 knockdown group, indicating a decrease of 62.6 ± 5.9% (*p* < 0.01, Figure 5B). These results confirm the significant role of TM7SF2 in the colony formation abilities of colorectal cancer cells.

## 4. Discussion

With advances in cancer diagnosis and treatment, the mortality rate of cancer has been decreasing. However, the survival rate remains low when metastasis occurs. This study aimed to identify biomarkers associated with cancer metastasis.

In this study, analyses using patient tissues and cell lines confirmed that TM7SF2 plays a crucial role in colorectal cancer. Immunohistochemical staining was used to assess TM7SF2 expression in patient tissues, including those from metastatic cases, revealing that TM7SF2 expression was higher in tumors at stages 3 and 4. Additionally, Kaplan–Meier analysis, chi-square analysis, and Cox regression analysis were conducted to evaluate the association between TM7SF2 expression and various clinicopathological factors. Kaplan–Meier analysis revealed a significant decline in the 5-year survival rate in patients with high TM7SF2 expression. Chi-square analysis demonstrated a significant increase in the proportion of patients with advanced clinical stages of colorectal cancer among those with high TM7SF2 expression. This finding suggests that TM7SF2 may play a crucial role in tumor progression and metastasis. Additionally, the significantly higher TM7SF2 expression in patients with advanced lymph node metastasis indicates that TM7SF2 overexpression may promote cancer cell invasion and metastasis. Furthermore, TM7SF2 expression was also elevated in cases with distant metastasis, suggesting that increased TM7SF2 expression may be associated with a more aggressive tumor phenotype in colorectal cancer. Cox regression analysis also demonstrated that higher TM7SF2 expression was significantly associated with decreased patient survival, suggesting that TM7SF2 may serve as an independent prognostic factor in colorectal cancer.

A previous study reported that TM7SF2 suppression inhibited the C-Raf/ERK1/2 pathway in cervical cancer [15]. This pathway plays a crucial role in promoting cancer progression [21,22,23,24,25]. In this study, TM7SF2 expression was silenced using siRNA in the colorectal cancer cell lines SW480 and SW620. The proliferation, migration, invasion, and colony-forming abilities of TM7SF2-silenced cells were compared with those of colorectal cancer cells with intact TM7SF2 expression. As a result, a significant decrease in cell proliferation, migration, invasion, and colony-forming ability was observed in the TM7SF2-silenced group. This finding suggests that the suppression of TM7SF2 expression may inhibit tumor progression and metastasis in colorectal cancer. Future studies should further investigate the functional changes and underlying mechanisms associated with reduced tumor progression following TM7SF2 suppression. Furthermore, it is necessary to investigate whether TM7SF2 suppression also inhibits the C-Raf/ERK1/2 pathway in colorectal cancer, as observed in cervical cancer.

## 5. Conclusions

In conclusion, the findings of this study highlight the potential of TM7SF2 as a metastatic biomarker and therapeutic target in colorectal cancer. Its significant association with cancer progression and poor prognosis underscores the need for further research into its functional mechanisms and therapeutic applications. A deeper understanding of TM7SF2’s role could lead to the development of more effective treatment strategies, particularly for patients with metastatic colorectal cancer or those for whom conventional therapies are less effective.

## Figures and Tables

**Table 1:** Samples of colorectal cancer patients used for immunohistochemistry.

Clinicopathological Factors		*N*
Gender	Male	164
	Female	72
pT stage	1	0
	2	24
	3	192
	4	20
pN stage	0	68
	1	84
	2	84
Metastasis	Negative	148
	Positive	88
* Clinical stage	I	8
	II	52
	III	84
	IV	92
Total		236

* Tumor stage was classified according to the American Joint Committee on Cancer tumor-node-metastasis (TNM) classification.

**Table 2:** Clinicopathological factors were associated with TM7SF2 expression in colorectal cancer patients.

Clinicopathological Factors	TM7SF2 Expression		Total	*p* Value
	Low (*N* = 104)	High (*N* = 132)	(*N* = 236)	
Age, years, mean (SD)	58.8 (8.8)	52.3 (11.4)	55.2 (10.9)	-
Gender, *N* (%)	-	-	-	0.015
Female	23 (31.9)	49 (68.1)	72	-
Male	81 (49.4)	83 (50.6)	164	-
pT stage, *N* (%)	-	-	-	0.002
pT1, pT2	18 (75)	6 (25)	24	-
pT3, pT4	86 (40.6)	126 (59.4)	212	-
pN stage, *N* (%)	-	-	-	0.006
pN0	40 (58.8)	28 (41.2)	68	-
pN1, pN2	64 (38.1)	104 (61.9)	168	-
Metastasis, *N* (%)	-	-	-	0.004
M0	76 (51.4)	72 (48.6)	148	-
M1	28 (31.8)	60 (68.2)	88	-
* Clinical Stage, *N* (%)	-	-	-	0.004
Stage I, II	36 (60)	24 (40)	60	-
Stage III, IV	68 (38.6)	108 (61.4)	176	-

* Tumor stage was classified according to the American Joint Committee on Cancer tumor-node-metastasis (TNM) classification.

**Table 3:** Univariate analysis of the clinicopathological parameters in patients with colorectal cancer.

Clinicopathological Factors	Variable	Univariate Analysis	
		Hazard Ratio (95%CI)	*p* Value
Age	≥60 yr vs. <60 yr	0.626 (0.455–0.862)	0.004
Gender	Female vs. Male	0.886 (0.644–1.219)	0.458
pT stage	T1-T2 vs. T3-T4	2.024 (1.124–3.643)	0.019
pN stage	N0 vs. N1-N2	1.962 (1.373–2.804)	<0.001
Metastasis	Negative vs. Positive	1.593 (1.164–2.179)	0.004
* Clinical stage	I, II vs. III, IV	3.862 (2.491–5.988)	<0.001
TM7SF2 expression	Low vs. High	2.128 (1.549–2.924)	0.001

* Tumor stage was classified according to the American Joint Committee on Cancer tumor-node-metastasis (TNM) classification.

**Table 4:** Multivariate analysis of the clinicopathological parameters in patients with colorectal cancer.

Clinicopathological Factors	Variable	Multivariate Analysis	
		Hazard Ratio (95%CI)	*p* Value
Age	≥60 yr vs. <60 yr	0.869 (0.580–1.303)	0.498
Gender	Female vs. Male	0.886 (0.620–1.268)	0.509
pT stage	T1-T2 vs. T3-T4	1.750 (0.887–3.452)	0.107
pN stage	N0 vs. N1-N2	0.490 (0.296–0.813)	0.006
Metastasis	Negative vs. Positive	0.838 (0.589–1.193)	0.327
* Clinical stage	I, II vs. III, IV	5.952 (3.277–10.812)	<0.001
TM7SF2 expression	Low vs. High	1.705 (1.205–2.412)	0.003

* Tumor stage was classified according to the American Joint Committee on Cancer tumor-node-metastasis (TNM) classification.

## Data Availability

The datasets used and/or analyzed during the current study are available from the corresponding author upon reasonable request.

## Supplementary Materials

The following supporting information can be downloaded at: https://www.mdpi.com/article/10.3390/curroncol32020114/s1, Full pictures of the Western blots in Figure 2.
