# Supplementary figures and images for: TM7SF2 as a Potential Biomarker in Colorectal Cancer: Implications for Metastasis
Source: Curr Oncol. 2025 Feb 17;32(2):114. doi: 10.3390/curroncol32020114 (PMC11854686; doi:10.3390/curroncol32020114)

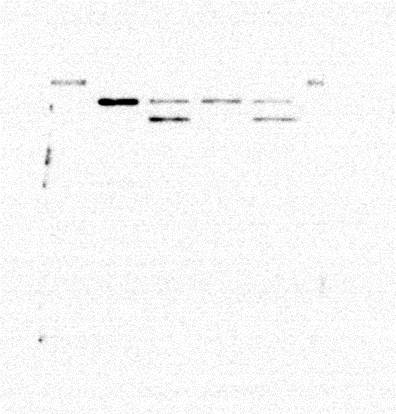

Supplement: Supplementary file 1 [file curroncol-32-00114-s001.zip › curroncol-3440152-supplementary/Blot image (1).tiff]

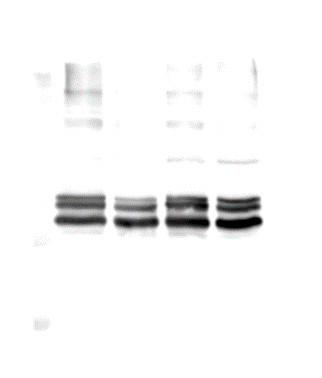

Supplement: Supplementary file 1 [file curroncol-32-00114-s001.zip › curroncol-3440152-supplementary/Blot image (2).tiff]
